# Evaluation of early African swine fever virus detection using CP204L gene encoding the p30 protein using quantitative polymerase chain reaction

**DOI:** 10.14202/vetworld.2024.1196-1201

**Published:** 2024-06-02

**Authors:** Ngo Thi Ngoc Tram, Danh Cong Lai, Do Thi Phuong Dung, Nguyen Tat Toan, Do Tien Duy

**Affiliations:** 1 Department of Infectious Diseases and Veterinary Public Health, Faculty of Animal Science and Veterinary Medicine, Nong Lam University, Ho Chi Minh City, Vietnam; 2 Nebraska Center for Virology, University of Nebraska, Lincoln, Nebraska 68583, USA

**Keywords:** African swine fever virus, diagnosis, immunohistochemistry, quantitative polymerase chain reaction

## Abstract

**Background and Aim:**

The African swine fever virus (ASFV), spanning 170–193 kb, contains over 200 proteins, including p72 and p30, which play crucial roles in the virus’s entry and expression. This study investigated the capability of detecting ASFV early through the analysis of genes B646L and CP204L, encoding p72 and p30 antigen proteins, by employing ASFV, diagnosis, immunohistochemistry (IHC), quantitative polymerase chain reaction (qPCR), and IHC techniques.

**Materials and Methods:**

Samples were taken from both experimentally and field-infected pigs to evaluate the effectiveness of qPCR and IHC in detecting ASFV. Twenty-two infected pigs were necropsied at 3-, 5-, 7-, and 9-day post-infection to obtain the first set of samples, collecting anticoagulated blood and tissues each time. The thymus, spleen, and lymph nodes were processed by fixing in 10% formalin, paraffin-blocking, and undergoing IHC staining. Forty anticoagulated blood samples were collected from clinically infected sows at a pig farm for the second batch of samples. Based on the lowest Ct values, three blood samples were diluted fivefold for qPCR DNA testing, and their tissues were used for both qPCR and IHC analyses.

**Results:**

At 1-day post-infection, p30-qPCR identified more ASFV-positive pigs and measured lower Ct values compared to p72-qPCR. At later time points, both methods showed similar levels of detection. ASFV was detected earlier and with lower Ct values in lymphoid tissues using p30-qPCR compared to p72-qPCR, particularly in the spleen and lymph nodes. In a field outbreak study, p30-qPCR demonstrated superior sensitivity and lower Ct values when detecting ASFV in blood samples compared to p72-qPCR.

**Conclusion:**

The early detection of the CP204L gene encoding p30 and its corresponding antigenic protein in ASFV diagnosis compared to the gene encoding p72 suggests that CP204L and p30 are promising candidates for the development of more effective antigen and antibody testing methods.

## Introduction

African swine fever (ASF) poses a significant global threat as a severe infectious disease to the swine industry. African swine fever virus (ASFV), belonging to the family *Asfarviridae* and genus *Asfivirus* [1], causes the debilitating disease. ASFV comprises both more than 60 structural proteins and over 100 non-structural proteins encoded in its double-stranded DNA genome [2, 3]. Several ASFV capsid proteins, including pp220, pp62, p72, p54, p30, and CD2v, play crucial roles in the virus’s attachment, entry, and replication processes [2]. The icosahedral capsid relies on the ASFV p72 protein encoded by B602L for its formation [4]. Meanwhile, the ASFV p30 protein, encoded by CP204L, can interact with heterogeneous nuclear ribonucleoprotein K during infection to downregulate host-cell mRNA translation [5]. Previous study by Cubillos *et al*. [6] have found that the recombinant p30 protein serves as a better diagnostic antigen than the p54 and p72 proteins. For serological investigations, the antigenic p30 protein is frequently utilized [7].

The World Organization for Animal Health provides various methods for detecting ASFV, including isolation, hemadsorption tests, polymerase chain reaction (PCR), loop-mediated isothermal amplification, enzyme-linked immunoassays, immunoblotting, and immunofluorescence assays [6, 8, 9]. Six days are needed for ASFV diagnosis through viral isolation, as it is the gold standard [10, 11]. Quantitative PCR (qPCR) assays are preferred for ASFV detection due to their efficiency, high sensitivity, and specificity [10, 12, 13]. Present-day qPCR tests primarily focus on the B602L gene, which encodes p72, as their target. The p30 protein is expressed earlier than the p72 protein during the early phase of virus replication, according to transcriptome analyses (3–5-h post-infection vs. 24-h post-infection) [14–16]. A recent study revealed distinct p30 and p72 protein expression levels during the early stages of ASF infection [17].

This study aimed to employ qPCR and immunohistochemistry (IHC) to diagnose ASFV early using genes CP204L and B646L and their proteins p30 and p72. The study’s findings may help detect ASFV earlier, improving pig farming disease control measures.

## Materials and Methods

### Ethical approval

The study was conducted in compliance with the institutional rules for the care and use of laboratory animals using a protocol approved by the Ministry of Agriculture and Rural Development Vietnam (TCVN 8402:2010).

### Study period and location

The study was conducted in the period from September 2019 to December 2020 through sample collection at a farm in a Southeast province, Vietnam and samples collected from an animal experiment at BSL3 animal facility of Vietnam National University of Agriculture and analyzed in the Raho6 laboratory.

### Animal experiments

Ten-week-old pigs (Twenty-two cross-bred) were orally infected with ASFV in the amount of 3 × 10^4^ 50% tissue culture infectious dose. In Binh Duong province, Vietnam, a highly virulent field strain (D/VN/BD/2019) was isolated from an infected pig in 2019. According to its p72 sequence, the strain matches genotype II with a perfect 100% identity to Georgia 2007. The virus multiplied in porcine alveolar macrophages and was quantified using the Reed–Muench method. Blood samples were taken from the test pigs at 1-, 3-, 5-, 7-, and 9-day post-inoculation. On each occasion, three to four pigs were randomly selected for necropsy to collect lymphoid tissues.

Forty whole blood samples were drawn from 40 pigs at an infected farm in Dong Nai province. Approximately 300 sows were raised at this farrow-to-finish farm.

### Sample collection and preparation

Animal whole blood was obtained from their jugular vein using K2 tubes (An Phu Jsc, Viet Nam). The test tubes containing the blood samples were stored at −20°C until required. Whole blood and tissue samples were processed for DNA extraction using the Wizard® genomic DNA purification kit (Promega, USA), according to the manufacturer’s instructions. qPCR utilized the extracted products stored at −20°C as raw material. Lymphoid tissues (thymus, spleen, and lymph nodes) from animals in experiment 1 were fixed using 10% formalin buffer. Thin sections were created from paraffin-embedded samples.

### Specific primers and probes

The sequences of primers and probes used to amplify p72 genes were used according to a previous study by King *et al*. [18]. Forward primer: 5′-CTG-CTC-ATG-GTA-TCA-ATC-TTA-TCG-A-3′; reverse primer: 5′-GAT-ACC-ACA-AGA-TC(AG)-GCC-GT-3′; and probe: 5′-FAM-CCA-CGG-GAG-GAA-TAC-CAA-CCC-AGT-G-3′-TAMRA. Meanwhile, p30-specific primers and probes were designed based on the nucleotide sequence of CP204L of the Georgia strain (accession no. FR682468.2) published in GenBank. The sequence of the forward primer is 5′-ATG-AAA-ATG-GAG-GTC-ATC-TTC-AAA-AC-3′; the reverse primer is 5’-AAG-TTT-AAT-GAC-CAT-GAG-TCT-TAC-C-3′; and the probe is 5′-FAM-5′-TGA-GCA-AGA-GCC-CTC-ATC-GGA-GGC-C-3′-BHQ1.

### The qPCR

A total of 20 μL p72-qPCR reaction mixture comprised of 10 μL SensiFAST™ Probe No-Rox master mix (Bioline, UK), 2 μL DNA template, 7 μL nuclease-free water, and 1 μL of a mix containing 42 μM forward primer, 33 μM reverse primer, and 25 μM probe. In a final volume of 20 μL, the p30-qPCR reaction contained 10 μL SensiFAST™ Probe No-Rox Master Mix, 2 μL DNA template, 6.5 μL nuclease-free water, 0.5 μL of each 5 μM forward and reverse primer, and 0.5 μL of 5 μM probe. qPCR amplification was performed using a Mic real-time PCR machine from Bio Molecular Systems in Upper Coomera, Queensland, Australia. 1 cycle at 95°C for 5 min, then 40 cycles at 95°C for 15 s, with a hold at 60°C for 40 s between each.

### IHC

The specific antibodies were used to detect and analyze ASFV p30 and p72 in serial sections of infected tissues. We employed rabbit polyclonal p30 antibody from Alpha Diagnostic Intl. Inc (San Antonio, Texas, USA) and mouse monoclonal p72 antibody from Ingenasa, Madrid, Spain as primary antibodies. Secondary antibodies were Dako’s polyclonal goat anti-rabbit and polyclonal goat anti-mouse immunoglobulins. The antigens, p30 and p72, were visualized using alkaline phosphatase staining for the former and diaminobenzidine staining for the latter. Ten fields (0.25 mm^2^) were randomly selected to quantify the number of ASFV antigen-positive cells from each IHC slide. The number of ASFV antigen-positive cells was ranked as follows: 0 = no positives, 1–10 = low positives, 11–30 = moderate positives, 31–100 = high positives, and >100 = very high positives.

## Results

**Figure-1:**
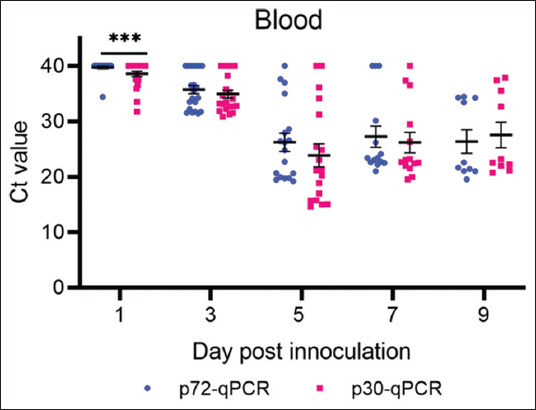
Comparison of Ct values of two quantitative polymerase chain reaction methods in blood at days after infection.

Twenty-two pigs inoculated with a virulent ASFV strain had their blood sampled at 1-, 3-, 5-, 7-, and 9-day post-infection. The two qPCR methods’ ASFV detection abilities were compared using the extracted DNA from these samples. At 1-day post-infection, only one pig tested positive for ASFV via p72-qPCR, with a Ct value of 34.42. Eight ASFV-positive pigs with Ct values ranging from 38.51 to 31.76 were detected by p30-qPCR. In the p30-qPCR assay, the Ct values were significantly lower than those in the p72-qPCR assay (p < 0.05). At 3-, 5-, 7-, and 9-day post-infection, there was no significant difference in positive pigs or viral load between Method A and Method B, as shown in Figure-1.

**Figure-2:**
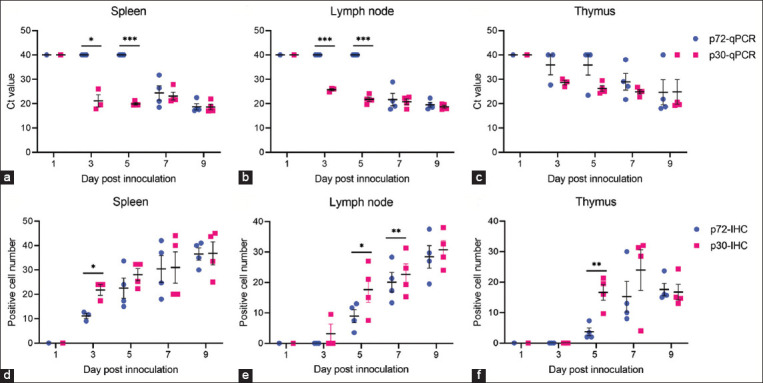
(a-f) Comparative analysis between quantitative polymerase chain reaction and immunohistochemistry in lymphoid tissues.

**Figure-3:**
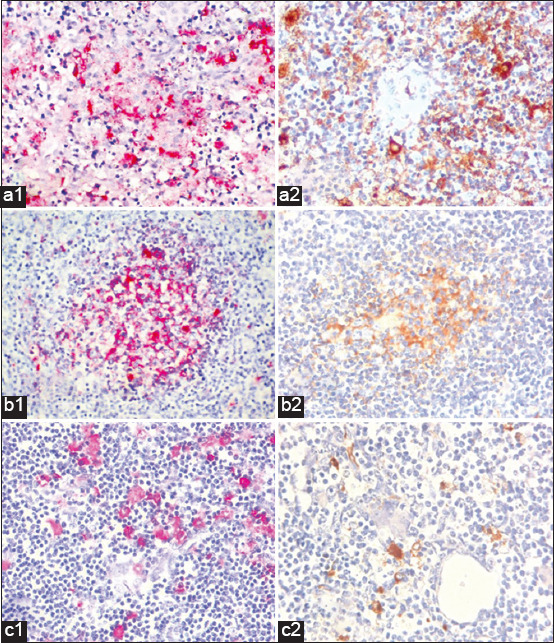
Positive immunohistochemistry images on (a) tissue sections of spleen, (b) lymph node, and (c) thymus from 5 dpi necropsied pigs. The positive signals of p30 (a1, b1, c1) and p72 (a2, b2, c2) were seen as red and brown grains, respectively.

At each time point, we randomly selected three to four animals for necropsy to acquire lymphoid tissues, such as spleen, thymus, and lymph nodes. The extracted DNA from the samples was utilized as a template for both techniques. At 3- and 5-day post-infection, we found substantial disparities in the Ct values of the two testing methods. One day post-infection, pig lymphoid samples tested negative for ASFV in both p72-qPCR and p30-qPCR. In the spleen and lymph nodes of three and four pigs at 3- and 5-day post-infection, ASFV was identified using p30-qPCR but not p72-qPCR (Figures-2a and b). In these samples, the Ct values obtained from p30-qPCR were significantly less than those derived from p72-qPCR. In Figure-2c, no significant disparities were detected in Ct values for thymus samples between the two methods. We also used IHC to assess and contrast the quantity of p72 and p30-expressing cells in lymphoid tissues. At the early infection stages, the number of ASFV-positive cells based on p30-IHC was significantly higher than that of p72-IHC (3 dpi in spleen, 5 dpi in lymph node, and 5, 7 dpi in thymus), as evidenced by Figures-2d–f and Figure 3, indicating the superiority of p30-qPCR over p72-qPCR in detecting ASFV.

**Figure-4:**
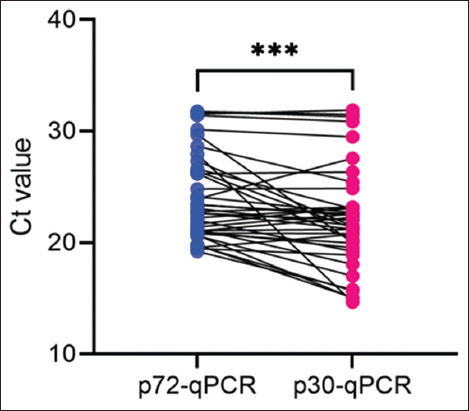
Comparison of Ct values of two quantitative polymerase chain reaction methods from the field samples.

We collected 40 blood samples during an ASF outbreak to validate p30-qPCR’s early detection ability. Samples were taken from sick pigs showing ASF symptoms such as refusal to eat, cyanosis, and lethargy. The presence of ASFV in DNA extracted from the samples was confirmed by both p72-qPCR and p30-qPCR methods. Both methods identified ASFV in all 40 samples (100% detection). Figure-4 shows the Ct values from p30-qPCR were significantly lower than those from p72-qPCR (22.14 ± 4.51 and 24.04 ± 3.84). p30-qPCR detects a higher viral load in the blood of ASFV-suspected pigs than p72-qPCR.

## Discussion

ASFV comprises more than 60 structural proteins in its complex structure. Both p30 and p72 play pivotal roles in viral entry, replication, and immune evasion [2, 19–21]. The p72 protein performs the dual role of contributing to the viral capsid’s structure and facilitating viral DNA replication. Meanwhile, the p30 protein, located in the inner membrane of the viral envelope, is one of the most immunogenic proteins [22, 23], which can induce a high level of viral antibodies, especially neutralizing antibodies [7, 19, 20]. Based on p30 antibodies, several commercial test kits for detecting ASFV infection are under investigation [23–28]. According to Lithgow *et al*. [29], the expression of p30 protein can be found within 2–4 h after infection, contrasting with p72 protein, which can be typically detected 24 h after infection. We proposed that the gene for p30 protein replicates earlier than the gene for p72 protein. We created a qPCR assay specifically for detecting CP204L, the gene responsible for p30 protein production. The assay was assessed using samples from infected pigs and a farm known to harbor ASFV. The p30-qPCR assay was more effective than the p72-qPCR test in detecting ASFV at the onset of infection. Using IHC analysis on experimentally infected pigs, we found that the p30 protein was expressed earlier than the p72 protein. Preliminary research findings from qPCRs and IHCs suggest that the CP204L gene encoding p30 is generated and displayed early in ASFV’s replication process.

The high sensitivity of p30-qPCR for ASFV detection is essential for effectively identifying and responding to outbreaks by detecting initial infections. Multiple investigations have homed in on p30 as a potential marker for identifying ASFV infection at its onset. The p30 protein’s early appearance during ASFV infection and its crucial antigenic properties enable the generation of p30-specific antibodies [2, 29, 30]. A previous study showed that anti-p30 antibodies may be detected approximately 8–12 days after infection [31]. A recent study also discovered that the ASFV p30 protein is generated early during infection and is an attractive antigen candidate for serological detection in a recent investigation [32]. These results showed that early ASFV p30 protein expression is crucial for the effective detection of ASFV infections. Employing CP204L and its associated protein as diagnostic markers can significantly improve early intervention and control efforts during outbreaks, thereby containing the virus’ spread.

## Conclusion

The early detection of the CP204L gene encoding p30 and its corresponding antigenic protein in ASFV diagnosis compared to the gene encoding p72 suggests that CP204L and p30 are promising candidates for the development of more effective antigen and antibody testing methods. However, the scope of the study was only comparative testing performed on blood and lymphoid samples via quantitative PCR technique without ELISA and other types of tissue.

## Authors’ Contributions

DTD and NTT: designed the study. DTPD, NTNT, and DCL: performed the experiments and analyzed the data; and NTT, DTD, and DCL: Wrote the manuscript. All authors read, reviewed, and approved the final manuscript.
